# Clinical and socio-demographic determinants of community reintegration in people with spinal cord injury in eThekwini Municipality, KwaZulu-Natal province

**DOI:** 10.4102/sajp.v78i1.1631

**Published:** 2022-05-27

**Authors:** Estelle Buys, Thayananthee Nadasan, Ntsikelelo Pefile, Michael O. Ogunlana, Deshini Naidoo

**Affiliations:** 1 Private Practice, Durban, South Africa; 2 Department of Physiotherapy, Faculty of Health Sciences, University of KwaZulu-Natal, Durban, South Africa; 3 Department of Occupational Therapy, Faculty of Health Sciences, University of KwaZulu-Natal, Durban, South Africa; 4 Department of Physiotherapy, Federal Medical Centre, Abeokuta, Nigeria

**Keywords:** community reintegration, spinal cord injury, functioning, participation, Reintegration to Normal Living Index (RNLI)

## Abstract

**Background:**

Community reintegration is a major outcome of rehabilitation after the acute phase in people with spinal cord injury (PWSCI).

**Objective:**

To investigated clinical and socio-demographic factors determining community participation in PWSCI, living in the greater eThekwini Municipality, KwaZulu-Natal province.

**Method:**

Our quantitative, cross-sectional study had a convenient sample of 41 PWSCI. A trained interviewer obtained socio-demographic information using a structured questionnaire. Participants completed the Reintegration to Normal Living Index (RNLI). Descriptive statistics were used in summarising the data; inferential statistics, -a t-test and analysis of variance (ANOVA) assessed the association of clinical and socio-demographic factors with the extent of community reintegration. A multiple linear regression investigated the determinants of community reintegration with the alpha level set at *p* = 0.05.

**Results:**

Mean age of the participants was 41 years (s.d.: 10, range 25–66), with the majority (*n* = 32, 78%) being male. The mean RNLI score was 68% (s.d.: 22, range 24–100). Participants scored higher on the RNLI if they were male (mean difference [MD] 18%, 95% confidence interval [CI]: 2–34), were employed (MD 16%, 95% CI: 0–32), had a salary (MD 19%, 95% CI: 5–32) and had no muscle spasms (MD 14%, 95% CI: 1–27. Muscle spasms (*p* = 0.012, 95% CI: 3.85–29.05) and being female PWSCI (*p* = 0.010, 95% CI: −35.75 to −5.18) were significant negative predictors of community reintegration.

**Conclusion:**

Community reintegration may be influenced by socio-economic factors. Special interventions for muscle spasms and support for women living with spinal cord injuries may enhance community reintegration.

**Clinical implication:**

Therapists need to focus on community reintegration with female PWSCI and on returning to PWSCI to work as this was improved community reintegration.

## Introduction

Spinal cord injury is a life-altering event which is associated with a wide range of impairments in health and functioning that lead to limitations in activity and restrictions in participation (Kirchberger et al. 2010; Van der Westhuizen, Mothabeng & Nkwenika 2017). The International Classification of Functioning, Disability and Health (ICF) framework (World Health Organization 2001) succinctly describes the information on functioning and disability in people with spinal cord injury (PWSCI), indicating that contextual, environmental and personal factors influence the development of disability in PWSCI (World Health Organization 2001). Community reintegration is the key outcome of rehabilitation after the acute phase of injury, as the end goal of rehabilitation is to facilitate PWSCI to participate in their life roles (Mothabeng 2011; Njoki, Frantz & Mpofu 2007; Scelza et al. 2007). It is dependent on physical functioning and a number of interrelated factors, such as overcoming barriers in the social and physical environment (Cieza et al. 2010; Lysack et al. 2007; Tsai et al. 2017). These factors may include employment, mobility and transportation, family support and physical accessibility in the community (Cieza et al. 2010; Mothabeng 2011). Community reintegration for PWSCI is a measure of participation when seen in the context of the ICF (Mothabeng 2011).

Fekete and Rauch (2012) reviewed 25 studies and concluded that environmental factors were consistently found to be correlates of physical activity in patients with SCI, whereas personal factors (socio-demographics and psychological constructs) were weakly associated with physical activity in the SCI population. Menon et al. (2015) restated the popular opinion that physical functioning in PWSCI significantly relates to the status of neurological recovery. This can be appreciated as the neural system is the source of motor function or physical performance. In South Africa, studies by (Joseph et al. 2015; Njoki et al. 2007; Phillips, Braaf & Joseph 2018) reported on the SCI population in the Western Cape province. Factors such as a lack of education, inaccessible housing and transport were identified as barriers to reintegration, with less focus on personal factors such as socio-demographics and the state of neurological recovery.

There are no studies investigating the determinants or factors associated with reintegration by PWSCI in eThekwini, KZN. Anecdotal information suggests that even though PWSCI possess the ability to access rehabilitation services, there remains a problem with reintegration into their respective communities. The lack of studies exploring the ability of PWSCI to reintegrate into their KwaZulu-Natal communities led us to conduct our study to objectively assess the ability of PWSCI to reintegrate into the community using the Reintegration to Normal Living Index (RNLI). There is a need to understand the determinants or factors associated with community reintegration by PWSCI in order to better understand the priorities for community rehabilitation.

Our article thus reports on the clinical and socio-demographic determinants of participation in PWSCI in eThekwini Municipality, KwaZulu-Natal province of the Republic of South Africa.

## Method

Our study is reported according to the guidelines for ‘Strengthening the Reporting of Observational studies in Epidemiology’ (Cuschieri 2019). A quantitative, cross-sectional, analytic design was used to investigate community reintegration scores achieved by participants and to identify the determinants of community reintegration in PWSCI. Our study was conducted in the greater eThekwini municipality, which consists of 92 urban and peri-urban districts. The main spoken language is IsiZulu (69%) and English (27%) and 11 other languages. There are both public and private sector healthcare facilities in eThekwini Municipality, which offer rehabilitation services.

Eligible participants were selected to participate once they had met the inclusion criteria. Potential participants were identified through the database of PWSCI in South Africa and recruited via telephone and/or mail.

The main criteria for inclusion were having a medical diagnosis of a SCI (quadriplegia and paraplegia, complete, incomplete, traumatic and non-traumatic), residing in eThekwini, KZN, being 18 years or older and representative of the South African population at large in terms of sex, race and age and being discharged from acute SCI care 12 months prior to our study. Participants were able to communicate in English or Zulu and signed written informed consent for participation. Participants were excluded if they had other medical co-morbidities that could compound the level of community reintegration. Pre-SCI morbidity that affected the activities of daily living also excluded participants.

A trained interviewer obtained the necessary information from the participants using a structured questionnaire. The questionnaire was used to obtain information on socio-demographic data (age, sex, ethnic group, marital status, type of housing and source of income) and clinical data (injury status, rehabilitation and therapy attendance, occurrences of secondary health complications, region of injury and diagnosis).

We used the RNLI to assess the participants’ ability to reintegrate into their community. The RNLI is a self-reported questionnaire that assesses an individual’s satisfaction with his or her performance in life activities (Wood-Dauphinee et al. 1988). The RNLI assesses mobility, self-care, daily activity, recreational activity and family roles. It has a visual analogue scale (VAS), three-point and four-point scoring systems. The RNLI demonstrates high internal consistency and adequate interrater reliability (patient and significant other) (Hitzig et al. 2012; Van der Westhuizen et al. 2017; Wood-Dauphinee et al. 1988). It is responsive to changes in the clinical status of patients, particularly when the subscales of ‘Daily Functioning’ and ‘Perception of Self’ are considered. In terms of criterion validity, the index is somewhat related to work status and disease status. It also demonstrates construct validity, both convergent and discriminant, when assessed against a quality-of-life index and an index of psychologic well-being (Hitzig et al. 2012; Mothabeng, Eksteen & Westaway 2012; Tooth et al. 2003).

Participants completed the RNLI to measure the degree of community reintegration (Spinal Cord Injury Rehab Evidence 2013). The RNLI was self-administered by the participants and consisted of 11 statements, which address seven different areas: indoor, community and distance mobility; self-care; daily activities of work and school; recreational and social activities; family role(s); personal relationships and presentation of self to others. The participant scores the statement that most accurately depicts what they are currently experiencing in day-to-day life. Each statement is rated on a 10-point VAS, 1 indicating ‘no reintegration’ and 10 indicating complete reintegration (Bourget et al. 2018). The total score of the RNLI is then converted into a percentage to categorise the participants’ perceived community participation.

### Ethical considerations

Ethical clearance to conduct this study was obtained from the University of KwaZulu-Natal Biomedical Research Ethics Committee (No. BE262/17). Permission was also obtained from any stakeholders, gatekeepers and hospital management from eThekwini Department of Health to obtain access to patient information.

### Statistical analysis

Descriptive statistics were used to present the data. A test of normality was performed on the RNLI% scores using the Kolmogorov-Smirnov test with a resultant *p* = 0.14, showing that the data were normally distributed. Independent *t*-tests and analysis of variance (ANOVA) were used to compare the RNLI with subgroups of PWSCI. The SPSS 25.0-version software (IBM Corp. 2017) was used for data analysis. Subgroups of participants were based on their socio-demographic and clinical characteristics. A multiple-linear regression was used to investigate the determinants of community reintegration with RNLI% as the outcome variable, while socio-demographic and clinical variables were predictor variables. The *p*-value for addition to the model was < 0.05 and for removal from model *p* > 0.1. A *p* < 0.05 was considered to be statistically significant; confidence intervals (CIs) were calculated for the β-estimate.

## Results

Forty-one PWSCI who met the inclusion criteria participated in our study. The mean age was 41 years (s.d.: 10, range: 25–66). The majority (*n* = 32, 78%) were men and slightly over above half of the participants (*n* = 23, 56%) were of the IsiZulu ethnic group. Table 1 and Table 2 presents the socio-demographic and clinical characteristics of the participants.

**TABLE 1:** Socio-demographic and clinical characteristics of the participants (*n* = 41).

Variable	Distribution	Frequency	Percentage
Sex of PWSCI	Male	32	78
	Female	9	22
Age group (years)	20–29	5	12.2
	30–39	12	29.3
	40–49	16	39.0
	50–59	6	14.6
	60 and above	2	4.9
Marital status	Single	24	58.5
	Married	13	31.7
	Divorced	3	7.3
	Widowed	1	2.4
Educational status	Primary	12	29.3
	Secondary	13	31.7
	Tertiary	16	39.0
Housing type	Rural	14	34.1
	Urban	27	65.9
Transport type	Private	33	82.5
	Public	8	17.5
Source of income	Salary	14	34.1
	Disability grant	19	46.3
	Workman’s compensation	4	9.8
	Investments	2	4.9
	Pension	1	2.4
	Savings	1	2.4
Presence of dependants	Yes	26	63.4
	No	15	36.6
Employment before injury	Yes	14	34.1
	No	27	65.9
Employment after injury	Yes	33	80.5
	No	8	19.5

PWSCI, people with spinal cord injury.

**TABLE 2:** Aetiology, type of injury, rehabilitation and secondary complication of participants (*n* = 41).

Variable	Distribution	Frequency	Percentage
Cause of SCI	Traumatic	31	75.6
	Non-traumatic	10	24.4
Injury status	Complete	26	63.4
	Incomplete	15	36.6
Anatomic region of injury	Cervical	15	36.6
	Thoracic	17	41.5
	Lumbar	9	22.0
Rehabilitation received	Inpatient	33	80.5
	Outpatient	8	19.5
Adherence to therapy	Yes	11	26.8
	No	30	73.2
Hospital attended	Private	18	43.9
	Public	23	56.1
Pain	Yes	32	78.0
	No	9	22.0
Muscle spasm	Yes	24	58.5
	No	17	41.5
Pressure sores	Yes	28	68.3
	No	13	31.7
Urinary tract infection	Yes	29	70.7
	No	12	29.3
Bladder and bowel complications	Yes	38	92.7
	No	3	7.3

SCI, spinal cord injury.

The mean RNLI score for the participants was 68% (s.d.: 22, range 24–100). The mean score of ‘daily functioning’ was 53 (s.d.: 18), while the mean score of ‘perception of self’ was 21 (s.d.: 9). The association between the socio-demographic or clinical variables and RNLI scores is presented in Tables 3 and 4.

**TABLE 3:** Association between socio-demographic or clinical variable and Reintegration to Normal Living Index visual analogue scale % (*n* = 41).

Variable	Distribution	Mean	± Standard deviation	*p*
Sex				0.024*
	Male	71.72	± 18.64	-
	Female	53.49	± 26.60	-
Age group (years)				0.62
	20–29	77.19	± 21.15	-
	30–39	70.98	± 20.11	-
	40–49	66.09	± 21.99	-
	50–59	57.18	± 25.77	-
	60 and above	69.16	± 25.74	-
Marital status				0.361
	Unmarried	65.59	± 22.30	-
	Married	72.31	± 20.27	-
Educational status				0.938
	Primary	64.01	± 21.12	-
	Secondary	68.39	± 26.02	-
	Tertiary	66.20	± 19.45	-
Housing type				0.788
	Rural	66.43	± 21.18	-
	Urban	68.38	± 22.28	-
Transport type				0.468
	Private	67.26	± 22.57	-
	Public	73.84	± 15.40	-
Source of income				0.030*
	Salary	78.33	± 19.46	-
	Disability grant	65.71	± 21.10	-
	Other	53.91	± 19.46	-
Presence of dependants				0.781
	Yes	66.99	± 20.51	-
	No	68.98	± 24.22	-
Employment before injury				0.803
	Yes	68.14	± 20.70	-
	No	65.98	± 26.79	-
Employment after injury				0.022*
	Yes	78.33	± 19.46	-
	No	62.21	± 20.98	-

s.d., standard deviation.

* , statistical significance given a confidence interval of 95%.

**TABLE 4:** Association between aetiology, type of injury, rehabilitation and secondary complications and Reintegration to Normal Living Index visual analogue scale % (*n* = 41).

Variable	Distribution	Mean	± Standard deviation	*p*
Cause of SCI				0.208
	Traumatic	70.16	± 21.16	-
	Non-traumatic	60.15	± 22.55	-
Injury status				0.843
	Complete	67.20	± 21.76	-
	Incomplete	68.61	± 22.20	-
Anatomical region of injury				0.497
	Cervical	70.43	± 18.21	-
	Thoracic	62.95	± 24.72	-
	Lumbar	72.19	± 21.42	-
Rehabilitation received				0.901
	Inpatient	67.51	± 22.31	-
	Outpatient	68.59	± 20.07	-
Adherence to therapy				0.461
	Yes	63.53	± 25.52	-
	No	69.25	± 20.34	-
Hospitals attended				0.682
	Private	69.31	± 21.16	-
	Public	66.47	± 22.43	-
Pain				0.030*
	Yes	64.92	± 23.13	-
	No	77.65	± 11.40	-
Muscle spasm				0.040*
	Yes	61.92	± 22.39	-
	No	75.91	± 18.17	-
Pressure sores				0.527
	Yes	66.24	± 20.28	-
	No	70.91	± 24.94	-
Urinary tract infection				0.451
	Yes	66.05	± 20.71	-
	No	71.74	± 24.28	-
Bladder and bowel complications				0.158
	Yes	69.06	± 21.04	-
	No	50.65	± 26.84	-

s.d., standard deviation.

* , Significance at *p* < 0.05.

The sex of the participants (*p* = 0.024), source of income (*p* = 0.03) and employment status after the SCI incident (*p* = 0.022) were significantly associated with the reintegration to normal life by PWSCI. The presence of pain (*p* = 0.03) and muscle spasm (*p* = 0.04) were significantly associated with a lower RNLI score.

Table 5 illustrates the results of the multivariate analysis relating RNLI% to socio-demographic and clinical variables (predictor variables). The model accommodated only predictor variables that had a *p*-value of < 0.05. The presence of muscle spasm (*p* = 0.012, 95% CI: 3.85–29.05) and being a female PWSCI (*p* = 0.010, 95% CI: −35.75 to −5.18) were significant negative predictors of community reintegration (RNLI%). Hence, female PWSCI who had muscle spasm are significantly less likely to achieve community reintegration than male PWSCI who did not have muscle spasm.

**TABLE 5:** Determinants of community reintegration using multiple linear regression relating Reintegration to Normal Living Index % to predictor variables (*n* = 41).

RNLI%	Coefficient	Beta	*p*	Lower CL	Upper CL	VIF
Constant	80.44	-	0.000	41.66	119.21	-
Sex of PWSCI	−20.47	−0.39	0.010*	−35.75	−5.18	1.24
Presence of spasm	16.45	0.38	0.012*	3.85	29.05	1.19
Presence of pain	4.90	0.09	0.487	−9.28	19.09	1.07
Income category	−7.19	−0.24	0.382	−23.66	9.28	4.34
Employment status post-stroke	−2.23	−0.05	0.857	−27.06	22.63	4.31

CI, confidence interval; RNLI, Reintegration to Normal Living Index; VIF, variance inflation factor; PWSCI, people with spinal cord injury.

Model *R*^2^ = 0.402, *p* = 0.02, *f* = 4.701.

* , Significant at *p* < 0.05.

## Discussion

Our study investigated factors that predict community reintegration in PWSCI. The community reintegration scores were significantly higher among men who earn a regular salary as their source of income, who are employed, and who do not have pain and muscle spasms after the SCI incident. Our results suggest that community reintegration is significantly associated with being male, earning a salary, being employed after the SCI incident and the absence of pain and muscle spasms. Community reintegration scores were not significantly associated with levels of education, cause of SCI, injury status and other variables. However, some studies (Nizeyimana, Joseph & Phillips 2020; Nizeyimana, Phillips & Joseph 2022) have shown that the extent of community reintegration is significantly associated with the level of education and employment status. Employment and education outcomes are fundamental aspects of achieving high community reintegration and quality of life among spinal cord injuries (Nizeyimana et al. 2020, 2022). Studies carried out in other regions of South Africa show a positive association between employment and psychosocial integration (Nizeyimana et al. 2022). Level of education and employment status have been shown to have a direct association among the general public (Chen & Wu 2007); however, employment status is the variable of interest when considering the quality of life or reintegration to function (Imanishi et al. 2017). Hence, our findings suggest that having employment with a regular source of income is expected to improve socio-economic status and community reintegration.

Most of the participants were male, with a male-to-female ratio of 3.5:1, findings that are similar to studies conducted in other regions of South Africa with a high prevalence of traumatic spinal cord injuries (Joseph et al. 2015; Pefile, Mothabeng & Naidoo 2018; Phillips et al. 2018). A study conducted in the same region found more women than men in non-traumatic spinal cord injury, which is attributed to the high incidence of retroviral disease in the region (Pefile et al. 2018). Sex is also associated with community reintegration, with women with spinal cord injury experiencing more barriers to all aspects of community reintegration than men (Forchheimer, Kalpakjian & Tate 2004). The needs of women with spinal cord injuries are often unmet, resulting in poor community reintegration outcomes (Jalovcic & Pentland 2009). Additionally, studies in developed countries have associated being male as a positive predictor of employment and education outcomes following spinal cord injury (Clark & Krause 2017).

Our study’s most affected age range is 20–39 years, with the overall mean being 41 years. These individuals are unlikely to participate in our study setting’s open labour market, resulting in poor community reintegration outcomes. There was no statistical association between age and community reintegration; however, the community reintegration mean scores were higher in this age group. Evidence from developed countries has shown that having sustained a spinal cord injury at a young age is associated with improved employment and education outcomes among persons with spinal cord injuries, often resulting in enhanced community reintegration (Marti et al. 2016; Rowell & Connelly 2010). However, the number of older PWSCI is growing, and there is a paucity of studies addressing community reintegration in this cohort in South Africa.

Most participants were unmarried; however, community reintegration scores were higher among married participants, although there was no statistical association. Studies have shown that being in a relationship or married at the time of injury is associated with high prospects of participation in economic activities that improve community reintegration. Access to housing type and transport are environmental factors that were not associated with community reintegration. These findings contrast with a study where the living conditions were related to community reintegration. It was shown that having access to houses in an urban setting improves community reintegration in PWSCI (Nizeyimana et al. 2022). Although there was no association between community reintegration and access to transport, an ability to independently use transport has been shown to improve social participation (Hwang et al. 2014; Tsai et al. 2017). Widening access to public transport and modified private vehicle use should be included in pursuits of community reintegration for PWSCI.

Both the presence of pain and muscle spasms were significantly associated with lower RNLI scores 64.92 ± 23.13 and 61.92 ± 22.39, respectively. This may be indicative of the fact that the participants who experienced pain and muscle spasms had more restrictions with community reintegration. Pain and muscle spasm have been observed to have negative effects on the quality of life (Mashola & Mothabeng 2019; Øderud 2014). It was interesting to note that neither pressure sores, urinary tract infections nor bowel and bladder complications resulted in a significant lowering of RNLI mean scores. Our result is contrary to other studies (Mothabeng 2011; Øderud 2014); however, one of the Australian studies reported that participants view health complications as a bump in the road, and as a result, these factors do not significantly influence community reintegration (Barclay et al. 2016). Individuals who sustained traumatic spinal cord injury with incomplete lesions had higher mean scores of community reintegration. The absence of secondary health conditions is also associated with higher community reintegration, which means scores with pain and muscle spasms have a significant association with community reintegration. The presence of secondary health conditions limits the individual’s ability to perform activities of daily living and participation in life situations (Mashola, Korkie & Mothabeng 2022; Pilusa, Myezwa & Potterton 2019). Moreover, the presence of secondary health conditions among PWSCI affects participation in employment and education activities, resulting in poor community reintegration (Tsai, Graves & Lai 2014).

We identified the variables that are determinants of community reintegration. These variables are both non-modifiable and modifiable factors that affect community reintegration among PWSCI. Non-modifiable factors include gender (being female) and are negatively associated with community reintegration. We have demonstrated the need for addressing gender inequality in managing community reintegration for PWSCI. Additionally, age and gender can be used to screen those individuals at risk of poor community reintegration outcomes during the rehabilitation process. The modifiable factor identified as a determinant of community reintegration for PWSCI is the presence of muscle spasms. This can be managed as a secondary health condition in sub-acute and chronic phases of rehabilitation following SCI. Interventions for the reduction in muscle spasms can be accessed via outpatient departments of secondary hospitals, specialised rehabilitation units or primary healthcare facilities. However, access to healthcare facilities among PWSCI in the study setting can undermine these efforts (Mlenzana et al. 2013). Return to normal functioning is expected to be affected by the presence of impairments; however, when PWSCI can access rehabilitation services, then disease clinical characteristics may not significantly affect community participation.

The RNLI is a self-reported measure, and it is subject to recall bias. We attempted to reduce this bias by providing assistance to the participants during data collection.

## Conclusion

There are significant associations among the socio-economic or pain status, normality of muscle tone in PWSCI and their level of community participation. When these variables are modified, functioning may be improved. Furthermore, we concluded that community reintegration among PWSCI is positively predicted by being male and the absence of muscle spasm. These factors are important for rehabilitation professionals to redirect inpatient and outpatient rehabilitation for optimal participation. It is, however, recommended that further research be conducted to objectively assess the extent of SCI disability and compare this with community participation.
